# Graphic complexity in writing systems

**DOI:** 10.1016/j.cognition.2021.104771

**Published:** 2021-09

**Authors:** Helena Miton, Olivier Morin

**Affiliations:** a Santa Fe Institute, 1399 Hyde Park Road, Santa Fe, NM 87501, USA; b Institut Jean Nicod, Département d'études cognitives, ENS, EHESS, CNRS, PSL University, UMR 8129, France; c Minds and Traditions Research Group, Max Planck Institute for the Science of Human History, Jena, Germany

**Keywords:** Graphic complexity, Visual complexity, Writing systems, Cultural evolution, Letters, Laterality

## Abstract

A writing system is a graphic code, i.e., a system of standardized pairings between symbols and meanings in which symbols take the form of images that can endure. The visual character of writing implies that written characters have to fit constraints of the human visual system. One aspect of this optimization lays in the graphic complexity of the characters used by scripts. Scripts are sets of graphic characters used for the written form of one language or more. Using computational methods over a large and diverse dataset (over 47,000 characters, from over 133 scripts), we answer three central questions about the visual complexity of written characters and the evolution of writing: (1) What determines character complexity? (2) Can we find traces of evolutionary change in character complexity? (3) Is complexity distributed in a way that makes character recognition easier? Our study suggests that (1) character complexity depends primarily on which linguistic unit the characters encode, and that (2) there is little evidence of evolutionary change in character complexity. Additionally (3) for an individual character, the half which is encountered first while reading tends to be more complex than that which is encountered last.

## 1 Introduction

Writing is a graphic code, i.e., a system of standardized pairings between symbols and meanings in which symbols take the form of images that can endure (Morin, Kelly, & Winters, 2020). It is a visual communication system which takes “the form of visible marks on the surface of a relatively permanent object” (Treiman & Kessler, 2011) and encodes a natural language (Morin et al., 2020).

Writing systems are based on characters organized into sets, here called scripts. Characters, in our definition, are the basic symbols used to visualize (write or print) a language. A script as we define it is a set of graphic characters used for the written form of one or more languages. A script is also sometimes one of several that are used for a language's writing system. Scripts, writing systems, and spoken languages rarely overlap perfectly. For instance, the Latin script is used by a variety of writing systems, to write a diversity of languages. Scripts do not determine what writing encodes, but they determine what writing looks like. For instance, the character ***a*** in the Latin script is defined by a basic shape which can be used in different languages to encode different sounds, depending on the context (language, word) in which it is used. Conversely, a writing system can be written using different scripts, e.g., Serbo-Croatian.

Written languages contrast with spoken languages. Writing is a relatively recent innovation: it can be traced back to a few invention events that occurred no more than a few thousand years ago. Writing requires, to a much larger degree than speaking, an explicit and deliberate effort in learning and transmitting it. While spoken languages extensively recruit auditory perception, written languages rely on the visual modality. This implies that written characters have to fit constraints of the human visual system (Dehaene, 2010; Dehaene & Cohen, 2007). At least two characteristics of writing systems reveal their adaptation to the human visual system. The characters of scripts are anisotropic with respect to the orientation of strokes within letters (preference for vertical and horizontal strokes over obliques), and of mirror symmetries (vertical symmetry being preferred to horizontal symmetry), two properties that can be predicted on neuroscientific grounds (Morin, 2018). They also tend to mimic natural scene statistics, by extensively using basic topological shapes that recur in the natural visual environment (Changizi, Zhang, Ye, & Shimojo, 2006; Testolin, Stoianov, & Zorzi, 2017), and cardinal orientations, which are overrepresented in the natural world (Morin, 2018). Both characteristics effectively reduce the cost of their processing by the human visual system. Another aspect of characters that determines their degree of adaptation to the human visual system is their visual complexity.

Visual complexity influences performance in reading and visual discrimination tasks (Donderi, 2006). Lower visual complexity correlates with easier learning, processing and use (Pelli, Burns, Farell, & Moore-Page, 2006). In addition to being easier to perceive, complex shapes arguably require more motor effort to produce, since they tend to involve a greater number of distinct strokes (distinct hand movements typically separated by a lifting of the inscribing instrument — Chang, Plaut, & Perfetti, 2016; Kim, Kim, Choi, & Kim, 1999; Rovenchak, Mačutek, & Riley, 2009). Simpler letters are easier on the eye and easier on the hand. Twin visual and motor pressures tend towards simpler letters.

### 1.1 Lexicon

**Script**: A set of graphic characters used for the written form of one or more languages, or the “graphic form of the units of a writing system” (Coulmas, 2002, p. 35). A script can be used by one or more writing system(s). For instance, the Latin script is used for several writing systems, including the English alphabet, or the Polish alphabet.

**Writing system:** A set of conventions linking a script to the sounds and words of a language. A writing system is usually based on one script only.

**(Writing system) type:** A way to categorize scripts based on the semiotics of the writing system they are used for, i.e., the linguistic unit their characters encode. Well known types include alphabets, syllabaries, logographies, etc. Here we often write “type” as short-hand for “writing system type”.

**Symbol/character:** We use the words “symbol” and “character” interchangeably. They refer to basic symbols (shapes) used to visualize (write or print) a language.

**Font:** A set of graphical representations of the shapes in a script. For instance, the font for the letters of the Latin script. This paper is not concerned with typographic variants, such as italic vs. regular type, or serif vs. sans-serif variants of a given script. We focus on the variation between scripts, which is considerable, rather than on variation inside scripts.

### 1.2 What determines the graphic complexity of a script's characters: Type, size, phylogeny?

Two main drivers of character complexity have been hypothesized: its graph inventory size, and the type of the writing system it is associated to (Chang et al., 2016; Changizi & Shimojo, 2005). The size of a script's inventory is the number of characters included in the script. For instance, the graph inventory size of the Latin [Latn]^1^ script is 52, as it includes 52 unique characters (we consider upper- and lower-case letters to be distinct characters). Writing system type is a way to categorize scripts based on the semiotics of the writing system they are used for, i.e., the linguistic unit their characters encode. Well known types include alphabets, syllabaries, logographies, etc. In addition to graph inventory size and writing system type, we add phylogenetic influence: the character complexity of one script can also be influenced by the script from which it descends. In other words, other things being equal, we consider the possibility that a script descending from a complex script may have more complex characters than a script descending from a simpler script.

#### 1.2.1 Size hypothesis: Scripts with larger graph inventories have more complex symbols

Studies investigating the relation between complexity and graph inventory size have yielded conflicting results. Changizi and Shimojo (2005) found that character complexity was of three strokes on average, independently of the script considered, while Chang et al. (2016) and Chang, Chen, and Perfetti (2018) found that character complexity increased with the number of characters included in a script, and was influenced by writing system type. Writing system type is, in this case, thought to influence the complexity of characters through the number of characters required by the mapping between characters and linguistic units. According to Chang et al. (2016), “the need for complexity is driven by the size of the grapheme inventory, which in turn is driven by the size of linguistic units to which they map: phonemes, syllables, syllabic morphemes, in increasing order” (p. 67).

One limitation of both studies is that they treat scripts as independent datapoints, even though distinct scripts are not independent. Scripts are related to other scripts. Many alphabetic writing systems originate from the Greek alphabet in more or less direct ways, for instance. Not accounting for common ancestry can be particularly problematic for cross-cultural data, as some of the characteristics observed in a population may be due to their common ancestry (“Galton's problem”): it is necessary to account for the fact that some observations are not independent from one another (Mace et al., 1994). Here, we want to test whether graph inventory size impacts the graphic complexity of characters, while accounting for possible influences from common ancestry.

#### 1.2.2 Homogeneity hypothesis: Most variance in character complexity is captured at the level of the script

Several arguments suggest that most of the variance in character complexity should be captured by knowing which script a character is from. First, inclusion in a given script captures many important sources of variance in character complexity that do not vary at the level of individual characters. This includes the material that the script is usually written on; the shape of the basic strokes making up the script; or general stylistic influences. Second, something like the principle of uniform information density (Jaeger, 2010), which guides spoken language production, may also constrain written language. If true, this would mean that writers maintain a more or less constant level of complexity throughout the various letters that they write. Third, similarity between characters increases predictability of features, which some have argued makes reading easier (Treiman & Kessler, 2011). Homogeneity between characters within a script also facilitates learning (Treiman & Kessler, 2011). For those three reasons, belonging to a particular script should be the most important factor affecting character complexity, when compared to the factors that are relevant to the complexity of individual characters —e.g., the type of writing system they are used for.

### 1.3 The cultural evolution of writing: Do characters become less complex over time?

Graphic complexity increases the cognitive cost of processing signals (Pelli et al., 2006). Graphic complexity also impacts the producer's effort, as more complex symbols take longer to draw or write, and are harder to reproduce faithfully (Tamariz & Kirby, 2015).

Higher visual complexity makes visual stimuli, and thus characters, harder to memorize and to recognize (Pelli et al., 2006; van der Helm, 2014) – see Section 2.3 for details on visual complexity metrics. More complex images (image here means any graphic representation, abstract or concrete, figurative or not) take longer to identify, and also occasion more mistakes, as they are more frequently confused with other symbols or reproduced imperfectly (Byrne, 1993; Donderi & McFadden, 2005; Pelli et al., 2006; Zhang, Zhang, Xue, Liu, & Yu, 2007). This effect of complexity is robust to participants' familiarity or experience with the images (Byrne, 1993), and to levels of noise, overall contrast, or eccentricity in the visual field (Shu, Chen, Anderson, Wu, & Xuan, 2003). Graphic complexity also weighs on the working memory load, making visual search harder (Alvarez & Cavanagh, 2004). Finally, the effects of graphic complexity occur early in the visual processing of words – earlier than orthographic or semantic effects (Dufau, Grainger, Midgley, & Holcomb, 2015). These results imply that a script can become more legible (up to a point) by decreasing its characters' complexity.

Complex drawings and scribbles are known to simplify in experimental settings, because simpler shapes take less effort both to remember and to produce. Drawings, in particular, have been among the first type of stimuli used in transmission chain experiments (Balfour, 1893; Bartlett, 1932). Transmission chain experiments function as games of “telephone”: one participant is given a stimulus that she has to reproduce. Her reproduction is then given to a second participant who has to reproduce it in turn, and so on until it reaches the last participant in the chain. Scribbles have been showed to decrease in complexity over experimental generations, especially when drawn from memory rather than directly copied (Tamariz & Kirby, 2015). Studies in experimental semiotics also suggest that written communication should show some form of compression. These experiments require one participant to guess a meaning among a set of possible options, based on drawings or scribbles produced by another participant. Whenever the same participants were allowed to play several rounds in a row, the drawings they produced became simpler, more abstract and less iconic (Garrod, Fay, Lee, Oberlander, & MacLeod, 2007). Scripts, during their lifetimes, are submitted to similar constraints: being reproduced from memory, transmitted, and used in communicative interactions. We would thus expect them to become simpler over time.

It has been suggested that changes in writing systems over time are relatively directed and that relatively iconographic or figurative variants (think Egyptian hieroglyphs [Egyp]) give rise to more abstract and simpler characters (Gelb, 1963). Iconic or figurative visual symbols tend to have more complex shapes than abstract symbols, as suggested by a study of hundreds of heraldic symbols (Miton & Morin, 2019). A case study focused on the Vai [Vaii] script showed that, at least for this recently invented script, characters indeed simplified during the two centuries that followed their creation c. 1833 (Kelly, Winters, Miton, & Morin, 2020).

#### 1.3.1 Invention hypothesis: Recently invented scripts are more complex than more ancient scripts

If pressures for simplification drive the evolution of scripts, we can expect that idiosyncratic scripts, i.e., scripts invented de novo by illiterate inventors in the recent past, with no overwhelming influence from one single ancestor, would have had less exposure to such pressures. In turn, this predicts that the characters of recently created scripts would be more complex than those of scripts that were exposed to evolutionary pressures for a longer period of time.

#### 1.3.2 Descendants hypothesis: Parent scripts have more complex characters compared to their offspring

Branching-out events occur whenever a script differentiates from its parent script: a large share of scripts were formed by branching out from other scripts. They did so either as independent offshoots of continuing scripts (Thaana [Thaa] from Arabic [Arab]), or as continuations of extinct scripts (Tifinagh [Tfng] or Greek [Grek] from Phoenician [Phnx]). Such branching-out events provide the opportunity to increase a script's efficiency, by simplifying its characters. If branching-out events favored an increase in efficiency, this would predict that the characters of the “parent” script would, on average, be more complex than their offspring's characters.

### 1.4 Order hypothesis: The distribution of complexity inside characters follows writing and reading direction

Studies of the Latin script suggest that visual complexity is not homogeneous within its letters. Latin letters tend to be “right-facing” (Fischer, 2011; Treiman & Kessler, 2011): most of their features are concentrated on the left hand-side half of the letter, as in capitals R, C, F, K, or E (Treiman & Kessler, 2011), so that they seem open to the right. Children pick this property of the Latin script early on, and commit mirror-writing mistakes more often when writing a left-facing character or digit (e.g. they turn “3” into “Ɛ” more often than they turn “K” into “ꓘ”) (Fischer, 2011, Fischer, 2013). In doing so, they transform characters in a way that puts their more complex half first (in reading and writing order) while retaining all their other characteristics. McIntosh, Anderson, and Henderson (2018) generalized this effect to pseudo-letters that children had no prior exposure to. This bias in letter production can be interpreted in several ways. Earlier studies claimed that Latin letters are right-facing because their right half contains more information compared to their left half, implying that the right-side half of letters contains more information than their left-side half (Kolers, 1969; Shimron & Navon, 1981). Yet the exact opposite conclusion can also be drawn: Latin letters appear right-facing because most of their graphic features (or strokes) are concentrated on the left side (as in E, F, P, etc.).

Recent work argues that the distribution of information inside Latin letters is biased towards the left half—the first half in reading and writing order. In line with this, Soares, Lages, Oliveira, and Hernández (2019) had Portuguese subjects complete a masked priming go/no go task, showing that words including mirror symmetrical letters (in this study, b or d) are processed more slowly when the letter's stem is oriented to the right: d takes more time to be processed than b, even though it is a much more frequent letter in Portuguese. The authors interpret this result as reflecting a “front-end coding scheme” whereby readers of the Latin script (as used in Portuguese writing) prioritize the information found in the front (i.e., left) part of letters. Concordant evidence comes from another script that, like the Latin script, is customarily (though not exclusively) written and read from left to right: Chinese characters. Using the chimeric stimuli paradigm, Janet Hsiao and her team show a left-side bias for Chinese character recognition (Chung, Liu, & Hsiao, 2017; Hsiao & Cottrell, 2009; Liu, Yeh, & Hsiao, 2018; Tso, Au, & Hsiao, 2014): the left side of characters carries a greater amount of distinctive information, compared to their right half. This effect replicates the well-known left-side bias obtained with other visual stimuli, like faces or dot patterns (Voyer, Voyer, & Tramonte, 2012).

The contradictory nature of claims and evidence on the distribution of visual information among letter halves is partly due to the lack of a clear and robust metric for visual information. Graphic complexity, as measured here, can (in our view) be used as a proxy for visual information (Pelli et al., 2006), providing us with a way of adjudicating between various views of the allocation of visual features between letter halves. We aimed to test the hypothesis that appears dominant in the literature: a concentration of visual information in the letter half that is written or read first (Chung et al., 2017; Hsiao & Cottrell, 2009; Tianyin Liu et al., 2018; Soares et al., 2019; Tso et al., 2014).

With rare exceptions (e.g. early Sumerian writing) reading and writing directions tend to coincide, and the direction of inscription tends to be the same between letters and inside letters: if letters are written from left to right, then the strokes inside these letters also tend to be written from left to right. There is good evidence that the strokes written first are more important in identifying a letter than those written last, at least for Chinese characters (Flores d'Arcais, 1994) and Latin letters (Parkinson & Khurana, 2007; Schubert, Reilhac, & McCloskey, 2018), consistent with the view that letter recognition recruits motor schemas supplementing purely visual memory (Perfetti & Harris, 2013). This could explain why most studies find evidence for a “front-end coding scheme” (Soares et al., 2019) where useful information is located first in reading and writing order.

We expected to find that letters in scripts written and read from left to right (like Latin) are “left-heavy”: their left half would be more complex than their right half. Conversely, letters in scripts written from right to left should be found to be “right-heavy”. When testing this prediction, we must control for a possible tendency for visual information to concentrate on the left or right side of characters, independently of reading direction. Visual perception has inbuilt asymmetries between the left and right visual hemifields, at least for some domains such as face perception (e.g. Voyer et al., 2012). Whether such an asymmetry applies to the perception of written characters independently of reading direction is, however, far from clear. Some studies suggest that information presented in the right-hemifield is given more weight (Hardyck, Tzeng, & Wang, 1977), while others find the opposite effect (Śmigasiewicz et al., 2010 for Latin characters, Chung et al., 2017 or Zhou, Gao, Chang, & Su, 2019 for Chinese characters). We nevertheless controlled for laterality, as a precaution.

We pre-registered two predictions based on the hypothesis that visual information clusters on the side of characters that the reading eye encounters first. Visual complexity should be higher in character halves that come first in reading order: left for Left-right scripts (i.e. scripts customarily written and read from left to right), right for Right-left scripts. Our second prediction was that complexity differentials between halves are specifically predicted by order: left halves are not more complex than right halves when they come last in reading order, and vice-versa.

## 2 Methods

### 2.1 Pre-registration and data accessibility

We kept a complete research diary on the Open Science Framework website (https://osf.io/9dnj3/) where all analyses carried out were pre-registered and described. Pre-registration consists in describing both the research design and analysis plan as independently as possible from data collection. Data and R scripts used to produce the results and figures can be found at https://osf.io/9dnj3/.

### 2.2 Inventory constitution

#### 2.2.1 Script-level inclusion rules

The inventory of scripts included in our study was compiled from the Unicode 10.0, updated according to the Unicode 11.0 (*The Unicode Standard, Version 11.0, (Mountain View, CA: The Unicode Consortium,* *The Unicode Standard, Version 11.0,* 2018*. ISBN 978–1–936,213-19-1)*, and enriched with official proposals to encode new scripts not included in the standard, but under consideration at data collection time (November–December 2018). This study excluded the following scripts. Secondary scripts, defined by Morin (2018) as scripts used by a writing system that encodes another system, e.g. Stenographics such as Duployan shorthand [Dupl]); non-visual scripts (e.g. Braille [Brai], a haptic script); scripts that do not directly encode a spoken language (e.g., Blissymbols [Blis]); and undeciphered (or only partially deciphered) scripts (e.g., Linear A [Lina]). Further exclusions occurred during data collection. Because our study required us to generate pictures of each character for each script, scripts for which we could not find a font (necessary to generate the pictures) were excluded. Finally, a symbol was considered missing if we could not produce a picture for it (i.e., if the font for the script did not have it). Scripts with up to 5 missing symbols were included. See the InventoryScripts.csv, in Files, at https://osf.io/9dnj3/ for an exhaustive list of exclusions and the justification for each.

#### 2.2.2 Character-level inclusion

Drawing on Morin (2018), a character was included if it could be used on its own by a writing system to encode one sound or (in the case of logographic systems) one word or phrase. We thus exclude the following: punctuation marks and ligatures, diacritic marks, number symbols, honorific marks, and currency marks. While diacritic marks encode sounds, they do not do so on their own and need to be associated to another letter, which is why we excluded them. The exclusion of ligatures and diacritic marks implied that the size variable (i.e., the number of characters included in a script) was to a small extent underestimated for abugidas and abjads (and their characters' average complexity overestimated, as diacritics and ligatures tend to be very simple), compared to syllabaries and alphabets. It is possible to account for the complexity of diacritics and their interactions with other characters' complexity when using different types of measures of complexity - for instance, by using the number of discontinuous elements in graph formation, as in the GraphCom dataset (Chang et al., 2018).

#### 2.2.3 Description of the dataset

**Fig. 1:**
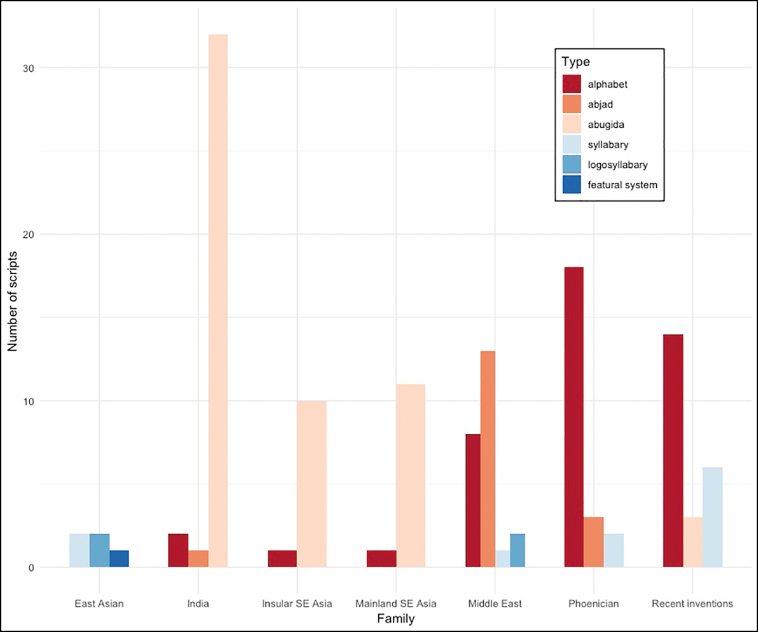
Composition of the dataset, by family and type of writing systems.

Our dataset was large and diverse: it included 47,880 characters from 133 scripts, comprising (see Fig. 1) 5 East Asian scripts, 23 Phoenician (European) scripts, 35 Indian scripts, 24 Middle East scripts, 23 Modern Inventions, 11 South East Asian Insular scripts, and 12 Mainland South East scripts. As for type of writing system, 17 abjads, 56 abugidas, 44 alphabets, 1 featural system,^2^ 4 logosyllabaries (morphosyllabaries), and 11 syllabaries were included.

#### 2.2.4 Dataset restrictions for the distribution of complexity inside characters

The set of scripts included for testing the prediction that characters' first halves are more complex than their last halves was the same as for the other hypotheses, with one exception. The scripts typically written and read in a top-to-bottom or bottom-to-top direction were excluded, along with scripts for which the direction of writing is uncertain, variable, or in boustrophedon style. The resulting list contains 124 scripts (97 scripts usually written from left to right, 27 scripts usually written and read from right to left). See https://osf.io/pmr34/ and detailed reports in Files at https://osf.io/v8khp/.

### 2.3 Measures of visual complexity

Following previous studies in cultural evolution (Kelly et al., 2020; Miton & Morin, 2019; Tamariz & Kirby, 2015), two measures of visual complexity were used, here called “perimetric” and “algorithmic”. Algorithmic and perimetric complexity measures were highly correlated, r_s_ = 0.87, *p* < 0.001 (as measured on pictures of full characters).

#### 2.3.1 Perimetric complexity

Perimetric complexity is defined as the ratio of inked surface to the perimeter of this inked surface (Attneave & Arnoult, 1956). It is obtained, using Watson's implementation (Watson, 2012), by taking the squared length of the inside and outside perimeters of a motif P, divided by the foreground area A and by 4π (Pelli et al., 2006; Watson, 2012):

$$C=\frac{P^{2}}{4\mathrm{\pi A}.}$$

The measure was implemented in Wolfram (Mathematica) (Watson, 2012). Perimetric complexity so defined correlates with human performance in learning to recognize and in discriminating letters (Liu, Chen, Liu, & Fu, 2012; Pelli et al., 2006).

#### 2.3.2 Algorithmic complexity

Algorithmic complexity measures are obtained by compressing the image file corresponding to a character (.eps file outputted by the Potrace algorithm). Algorithmic complexity is then the size in bytes of the compressed file: it offers an estimation of the length of the shortest computer program that produces the picture of the character without loss of information. This measure correlates well with perimetric complexity (Miton & Morin, 2019).

### 2.4 Pictures processing

Our analyses required a standardized collection of pictures, in which the amount of variation due to the use of different fonts would be minimized, while the variation due to actual character shapes would be preserved. Among other things, fonts vary on two properties that can affect the measures of character complexity: size and line thickness.

#### 2.4.1 Generating pictures of characters

A picture of each character was generated using a range of Unicode identifiers (an identifier is a code of four or five alphanumeric characters uniquely identifying a character) and a font (a font is a particular graphical instantiation of a character). The script generating the pictures (written in bash) fixed the size of the picture at 500 by 500 pixels, and an initial font size for drawing the symbols at 60. Whenever a script presented characters that would be to too big to fully fit within the 500 by 500 pixels canvas, it was rerun at a smaller point size. In such cases, we decreased point size 5 by 5, until reaching a size at which all characters would fit inside the canvas. This was necessary for only four scripts ([Egyp], [Bali], [Mymr], [Gran], with respective final point sizes of 55, 45, 55 and 40).

#### 2.4.2 Resizing

**Fig. 2:**
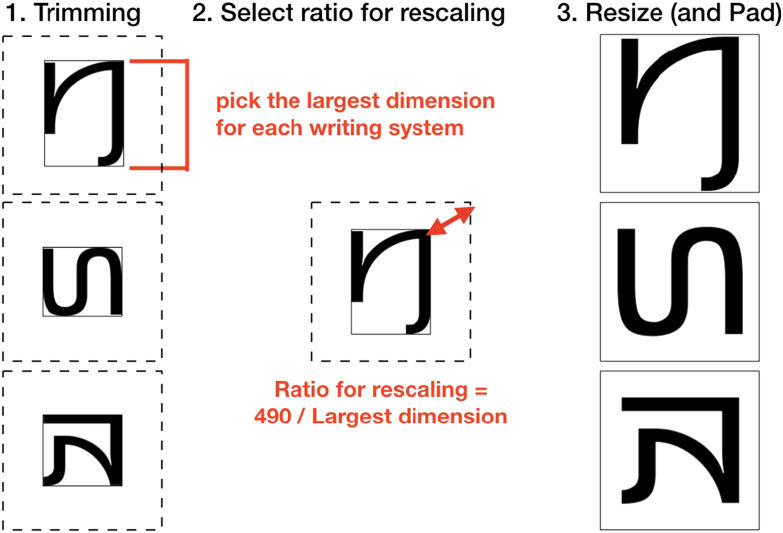
Procedure used to minimize the variation in characters' size between scripts. This example uses characters (from top to bottom) A91A, A91B and A90F from the Kayah Li [Kali] script.

In order to standardize our pictures for size across scripts, we adaptively resized them. We first trimmed all the pictures. We then selected, for each script, the character with the largest picture (on either dimension, i.e., height or width). From this picture, we derived a ratio specifying how much it had to be resized for its largest dimension to fit a 490 by 490 pixels square (maintaining the aspect ratio and thus avoiding distortions). Finally, we used this ratio for resizing all pictures from the same script, and placed the resulting pictures back on a 500 by 500 pixels white canvas—see Fig. 2. This procedure allowed us to minimize variation in size between different scripts, even when they used very different fonts, while maintaining the variation in size occurring *within* each script.

#### 2.4.3 Homogenizing line thickness

**Fig. 3:**
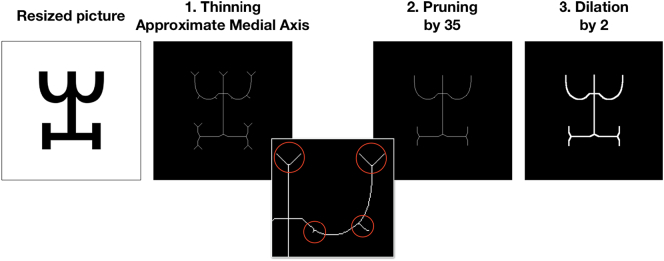
Procedure used to minimize the variation in line thickness, both within and between scripts. The red circles show examples of small problematic strokes appearing during the thinning step. This example shows the character A607 from the Vai [Vaii] script. (For interpretation of the references to color in this figure legend, the reader is referred to the web version of this article.)

In order to obtain a collection of characters with the same constant line thickness, we used a combination of functions in Mathematica: first thinning, then pruning, and finally, dilation (See Fig. 3.). The Thinning function (argument “Method” set on “MedialAxis”) returned the approximate medial axis of the picture. Then, we applied a Pruning function (argument = 35) in order to eliminate some of the artefacts emerging from the process of obtaining the approximate medial axis. This effectively removed the small segments that appeared during the extraction of the approximate medial axis but were not part of the optimal (i.e., representative) skeleton of the character. Pruning branches whose length was inferior to 35 pixels yielded satisfactory results. Finally, the Dilation function (argument = 2) made the trait thicker and more akin to usual characters. This procedure resulted in white characters on a black background (on which perimetric complexity measures were computed in Mathematica).

#### 2.4.4 Additional treatment for algorithmic complexity

Algorithmic complexity metrics were computed on pictures having a black foreground (black character) over a white background. Each character's picture also went through the Potrace algorithm (Selinger, 2003), to eliminate any superfluous pixels and to obtain a vectorized version before zip compression.

#### 2.4.5 Pictures of characters' vertical halves

Each character picture in our dataset was automatically split vertically, i.e., it was cut into two halves (right and left) separated by a vertical line.

**Fig. 4:**
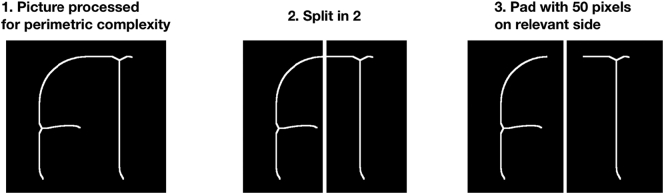
(1) We start with pictures as processed for perimetric complexity (i.e., white on black, pnm format, square pictures of 500 by 500 pixels). (2) These pictures are split vertically into two halves (of 250 by 500 pixels). (3) They are padded on the side on which the character is cut – i.e., the “inner side” of the initial picture, to a final size of 300 by 500 pixels. All other steps required for the pictures to be fitted for algorithmic complexity measurement were then applied (i.e., reversing colors so that the character is in black on a white background, applying the potrace algorithm for vectorization). This figure illustrates the process by using character 1E90F from the Adlam [Adlm] script.

To do so, we used the pictures exactly as they had been processed as described in the preceding steps. We split them in two equal-size halves, and re-padded them to an equal, constant size (see Fig. 4). Padding avoids having parts of the characters starting at the picture's border, which can be problematic for calculating the perimetric complexity measures in Mathematica. Padding here means that we add pixels of the background's color (black) on the side that directly cuts the character (see step 3 of Fig. 4). Vectorized pictures cannot easily be cut in half because they do not have a ‘size’ in pixels, so the cutting step was applied on pictures processed for perimetric complexity, and then transformed again to compute algorithmic complexity measures.

### 2.5 Phylogeny, size, type, and other information

The variables included in our analyses were the graph inventory size of each script (the number of characters that it includes), its family (a category based on each script's geography and ancestry), its type (e.g., alphabet, abugida, syllabary, etc.), and whether or not the script was idiosyncratic. We classify scripts created by identifiable creators in the last two centuries, with no predominant influence from any single existing script, as “idiosyncratic”. For analyses using character-level measures, an additional grouping variable script refers to the specific script to which they belong. Whenever applicable, ancestor, i.e., which other script is considered an ancestor of the script, was also used.

#### 2.5.1 Sources

Our starting point was the dataset published in Morin (2018). To obtain information on scripts that were included in this study but not in Morin (2018), we used the sources listed in that paper. One of the sources used in Morin (2018) (“Ethnologue: Languages of the World” n.d.), could not be used in our study, due to its shift to a for-pay model. All variables were coded by pooling together all available information from our sources. A majority rule was applied whenever our sources gave contradictory information.

#### 2.5.2 Graph inventory size

Graph inventory size was measured as the number of unique characters included in our sample for each script. When a character exists in several possible versions depending on its position (e.g. capital letters vs. minuscules in the modern Latin script), we counted each version as one distinct character, following the Unicode Standard.

#### 2.5.3 Script classification: Families

Our script family variable mostly followed the classification established by Morin (2018) (drawing mainly on Daniels & Bright, 1996) on phylogenetic and geographic grounds. The seven families were the following:

- Middle Eastern family: direct descendants of the main scripts of the Middle East (i.e. Egyptian, Cuneiform, South Arabic, and Aramaic scripts).
- Phoenician family (“European family” in Morin, 2018): all the direct and indirect descendants of the Phoenician alphabet, including the Greek alphabet's script and its descendants.
- Indian Brahmic family: all the descendants of the Brahmic script in Modern India, Pakistan, Sri Lanka, Mongolia, and Tibet.
- Mainland South-East Asian Brahmic family: all the direct and indirect descendants of the Brahmic script in mainland South-East Asia.
- Insular South-East Asian Brahmic family: all the direct and indirect descendants of the Brahmic script outside of mainland South-East Asia, in Indonesia, and the Philippines.
- Recent inventions family: all the scripts created after 1800.
- East Asian family: Korean Hangul, Japanese Kanas, and Chinese scripts unrelated to the Brahmi script (i.e. Han [Hani], Yi [Yiii], Tangut [Tang]).

This family variable is not strictly phylogenetic: in addition to phylogenetic information in the form of ancestry (i.e., parent and offspring scripts), family also includes geographic information. This classification adheres to and reflects previously established conventions in reference documents seminal to the study of writing systems (Daniels & Bright, 1996). A strict interpretation of phylogenetic information is captured by our ancestor variable.

As for all other variables, each script's last common ancestor was determined by pooling together information from all our sources. When sources were consistent with one another but differed in their specificity, the most specific source (citing the ancestor that was closest in time to its descendant) was chosen.

#### 2.5.4 Types of writing systems

Based on definitions from Daniels & Bright (1996, p. 4), we classified scripts according to the linguistic unit their graphemes mapped onto, and recoded the information from our sources according to the following classification (all quotations below from Daniels & Bright, 1996):

- alphabet: “the characters denote consonants and vowels”.
- abjad (or consonantary in Daniels & Bright, 1996): “the characters denote consonants (only)”. In other words, such scripts let readers supply the appropriate vowel.
- abugida: “each character denotes a consonant accompanied by a specific vowel, and the other vowels are denoted by a consistent modification of the consonant symbols”. They are also referred to as syllabic alphabets, or alphasyllabaries in other sources.
- syllabary: “the characters denote particular syllables, and there is no systematic graphic similarity between the characters for phonetically similar syllables”.
- logosyllabary, or morphosyllabary: “the characters of a script denote individual words (or morphemes) as well as particular syllables”. Our dataset included four such scripts: Egyptian hieroglyphs [Egyp], Chinese characters [Hani], Anatolian hieroglyphs [Hluw] and Tangut [Tang].
- featural: “the shapes of the characters correlate with distinctive features of the segments of the language”. The only such script in our sample is Hangul [Hang].

#### 2.5.5 Idiosyncratic scripts

Scripts were considered idiosyncratic if they fulfilled the following criteria. (1) precise information is known about their inventors (most often, their name). (2) There is no scholarly consensus that they derive their shape from the influence of one single identified ancestor. Most resemble no known script; others fuse many influences together so that no single dominant influence is discernible. (3) The script was invented after 1800. This definition excludes invented scripts such as Cherokee [Cher], which was invented de novo by an identifiable inventor, but nonetheless bears the dominant influence of one script (in Cherokee's case, the Latin script).

#### 2.5.6 Direction of writing

Direction of writing was obtained from four sources — two websites: Wikipedia and Omniglot, and two reference books (Daniels & Bright, 1996; Rogers, 2005). We decided to use the dominant direction of writing in our coding, even for scripts that are occasionally written in the opposite direction. For instance, the sinogram-based script used for the Chinese languages [Hani] is usually written and read from left to right, but occasionally also from right to left: we nonetheless decided to code it as written and read from left to right. For all the scripts included in this study and mentioned in Morin, 2018 (*n* = 109) we used Morin's information after double-checking it against Omniglot. Scripts that were not referenced on Omniglot were double-checked with the ScriptSource website. For the remaining 24 scripts, a consensus method was applied.

For six scripts, we found discrepancies between Morin's coding and Omniglot (or ScriptSource if the script wasn't referenced on Omniglot). In two cases we established that Morin was mistaken (Meroïtic hieroglyphs [Mero] and Meroïtic cursive [Merc]). In four other cases, disagreements between sources were important enough to conclude that the script was either not well documented, or extremely variable, with regard to direction of writing. These scripts were thus excluded: the Batak script (for Karo) [Batk], the Japanese syllabaries [Hrkt], the Linear B script [Linb], and the Old North Arabian script [Narb].

## 3 Results

### 3.1 Size hypothesis

**Fig. 5:**
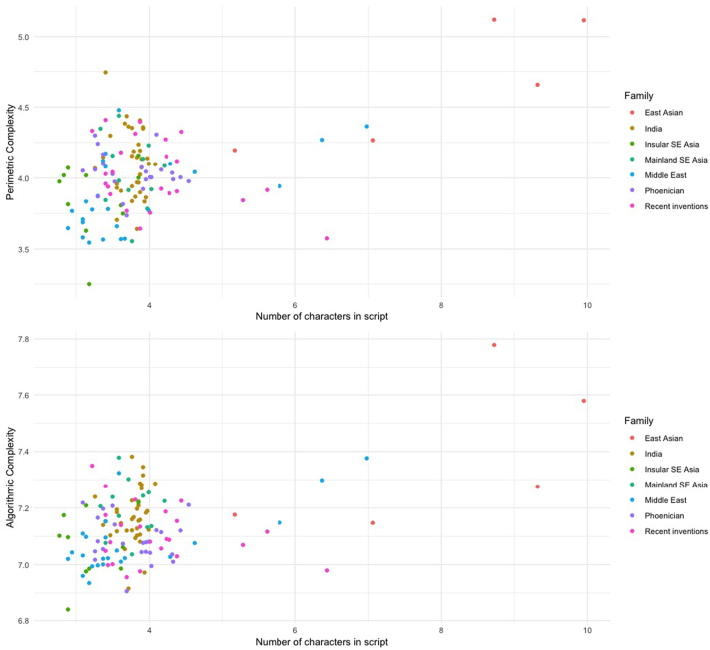
Script complexity (the average complexity of characters in a given script, perimetric above, algorithmic below) as a function of graph inventory size. Color shows script family. Both complexity measures and the number of characters in scripts were log-transformed.

Size has an impact on character complexity: the more characters in a script, the more complex the characters. However, this effect depended on whether large scripts (with graph inventory size >200), including logosyllabaries (or morphosyllabaries), were included or not (see Fig. 5).

The size variable (number of characters in a script) was used as predictor in a nested regression analysis. The scripts were grouped by family to account for shared cultural influences between distinct scripts. These families were used as the grouping variable in linear mixed models with random intercept, using the lmer function of the lme4 package for R (Bates, Maechler, Bolker, & Walker, 2014). A null model was built first, with a random intercept for family and for type; a second model introduced the script's size (i.e., graph inventory size) as a fixed effect.

On the full dataset (total *N =* 47,880 characters from 133 scripts), the best null model for characters' perimetric complexity included both type and script (the latter variable nested by family) as random effects. A model adding size as a fixed effect shows larger scripts to be more complex than smaller ones (β = 0.12, 95%CI[0.073, 0.175], df = 21.222, *t* = 4.78, *p* < 0.001 for perimetric complexity; β = 0.04, 95%CI[0.0245, 0.0747], df = 24.79, *t* = 3.873, *p* < 0.01 for algorithmic complexity). Models were refitted using maximum likelihood for comparison purposes, showing that the test model was more informative (informativeness being assessed using Akaike's Information Criterion, AIC) (Δ_AIC_ = 12.5 for perimetic complexity, Δ_AIC_ = 46 for algorithmic complexity).

Using a subset comprising exclusively scripts with less than 200 characters (*N =* 5566*,* on 124 scripts), similar to Changizi and Shimojo (2005), there is no longer an effect of graph inventory size. If we remove large scripts (scripts including 200 characters or more) from the dataset, larger scripts were not more complex than simpler ones, neither for perimetric (b = 0.06, 95%CI[−0.048, 0.168], df = 92.03, *t* = 1.086, *p* = 0.28) nor for algorithmic complexity (b = 0.03, 95%CI[−0.012, 0.076], df = 100.58, *t* = 1.42, *p* = 0.159).

Most of the effect of graph inventory size seems to depend on the inclusion of a few very large systems (mostly East Asian) which also tended to have very complex characters. We thus replicated the results from both Changizi and Shimojo's (2005) and Chang et al.'s (2016) results: character complexity does not seem to be influenced by size, as long as we restrict our analyses to the scripts in the same range as Changizi and Shimojo (2005)’s analyses. However, we show that this null result depends on the exclusion of high-size, high-complexity scripts. As all such large and highly complex scripts correspond to those which are logographic in our dataset, this finding is consistent with Chang et al.'s interpretation that type determines the size differentials that matter for character complexity.

### 3.2 Homogeneity hypothesis

**Fig. 6:**
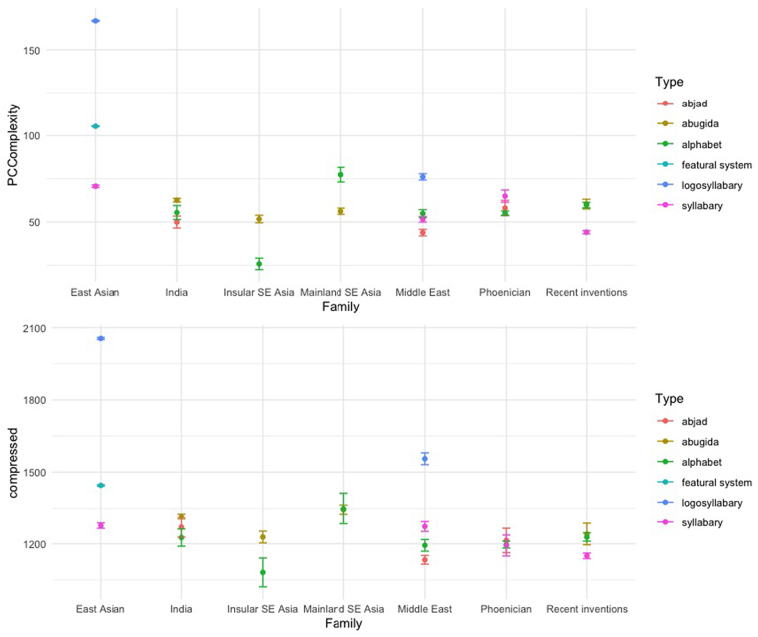
Complexity by family and type (error bars represent 95% confidence intervals): the top panel represents perimetric complexity, the bottom panel represents algorithmic complexity.

We predicted that the script to which a character belongs would account for over half of the variance in character complexity. Contrary to our prediction, the script variable accounted for less than half of the variance, and in fact type captured more of the variance in character complexity than either script or family. This was true for both perimetric and algorithmic complexity, see Fig. 6.

The intraclass correlation (ICC) was calculated on raw values for perimetric complexity measures and on log-transformed values for algorithmic complexity (in order to avoid convergence issues), using the ICC1.lme function in the “psychometric” R package (Fletcher, 2010). 38.57% of the variance in perimetric complexity and 38.49% of the variance in algorithmic complexity is accounted for by a character's inclusion in a particular script. By comparison, family accounts for 29.74% (algorithmic complexity) to 45% (perimetric complexity) of the variance, whereas type captures 68.26% of the variance in perimetric complexity and 55.43% of the variance in algorithmic complexity. Most of the variance in letter complexity is thus captured by the type of writing system which the letter belongs to (e.g., alphabetic, syllabic, etc.). The actual script that a letter belongs to (e.g. Brahmic [Brah], Greek [Grek]) does not predict as much variance as its type. This was extremely surprising to us, given all the things that letters from the same script share: a common history, a set of basic constituent strokes, a preferred medium of inscription, a population of users, etc.

### 3.3 Invention hypothesis

Our hypothesis predicted that characters from idiosyncratic scripts would be more complex than characters from non-idiosyncratic scripts. The null model for this hypothesis thus did not include family as a random effect: random effects only included type and script. Contrary to our predictions, adding the Idiosyncratic variable did not improve the model fit, for both perimetric and algorithmic complexity measures. The test model failed to show any effect of idiosyncratic (β = 0.016, 95%CI[−0.128, 0.161], df = 126.08, *t* = 0.226, *p* = 0.822 for perimetric complexity; β = −0.003, 95%CI[−0.062, 0.056], df = 125.05, *t* = −0.108, *p* = 0.914 for algorithmic complexity), when compared to the best null model for characters' complexity (Δ_AIC_ = − 2.0, for both perimetric and algorithmic complexity). Additionally, idiosyncratic scripts were neither more nor less complex than the other scripts from the Recent Inventions family that were *not* idiosyncratic (β = 0.68, 95%CI[−10.67, 12.79], df = 20.98, *t* = 0.11, *p* = 0.913 for perimetric complexity, β = 9.72, 95%CI[− 103.45, 121.56], df = 20.28, *t* = 0.172, *p* = 0.865, for algorithmic complexity, characters nested by script for both).

### 3.4 Descendants hypothesis

We hypothesized that, considering branching-out events, a “parent” script's characters would be more complex than its “offspring” script's characters. For each pair, the ancestor's average complexity (i.e., the mean complexity of its characters) was subtracted from the descendant's average complexity (as pre-registered). Our dataset included information on 102 branching-out events, from 29 different ancestor scripts. The most frequent parent script was Brahmi [Brah], with 25 offspring scripts. A parent script had, on average, 3.55 descendants (SD = 4.98).

**Fig. 7:**
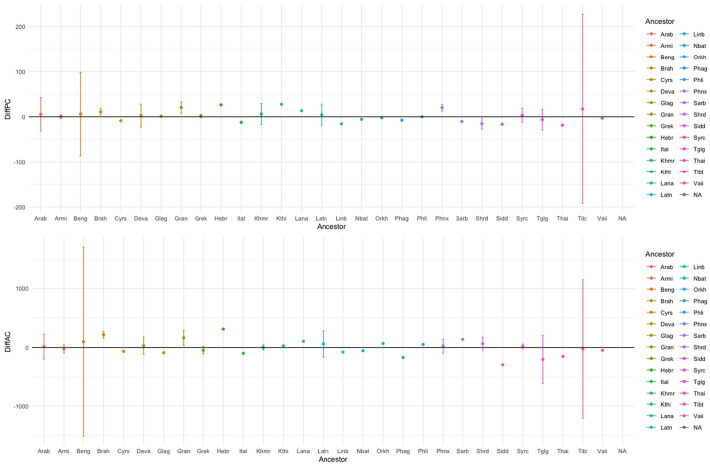
Average difference between means of descendant scripts and ancestor scripts plotted for each documented ancestor script, by alphabetic order (ISO key), for perimetric complexity (top) and algorithmic complexity (bottom). Error bars represent 95% confidence intervals. Order Hypothesis

When controlling for ancestry (i.e, including ancestor as a random effect), algorithmic complexity did not seem subject to any systematic effect: no significant increase or decrease in complexity occurred with branching-out events (β = 12.87, 95%CI [−34.98, 57.71], df = 29.69, *t* = 0.563, *p* = 0.577). Perimetric complexity tended to *increase* (not decrease) with branching-out events, but this trend failed to reach significance (β = 3.734, 95%CI [−0.65, 7.35], df = 21.44, *t* = 1.823, *p* = 0.082), see Fig. 7. These results suggest that the null hypothesis may be true (no tendency for descendants to diverge from ancestors in a particular direction, as far as complexity is concerned).

A Bayesian one-sample *t*-test was conducted to see whether the data supported the hypothesis that descendants do not, on average, decrease or increase in complexity, relative to their ancestor. It found moderate support for the null for both perimetric (BF = 4.02) and algorithmic complexity (BF = 5.06) – see Fig. 7. Differentials were averaged for each ancestor, rather than for each descendant-ancestor pair, to avoid assigning more weight to ancestors with numerous descendants (such as the Brahmi script).

#### 3.4.1 Prediction 1: First halves are more complex than last halves

We calculated, for each character and for both measures of complexity, the first-half / last-half differential, i.e., the complexity of the first half, minus that of the last half. The first half was the half coming first in writing order, i.e., left in a script written from left to right, right in a script written from right to left. Data points were individual characters, nested by script and by family. A significantly positive intercept, meaning that first halves are, on average, more complex than last halves, controlling for family or writing direction, would confirm our prediction. This proved true for measures of algorithmic complexity (intercept estimate = 16.050, 95% CI [8.971, 23.128]; *df* = 140.67, *t* = 4.444, *p* < 0.001). Adding the script's average character complexity to our model did not make it more informative (Δ_AIC_ = 6 in favor of the original model), suggesting the effect is evenly spread between high-complexity and low-complexity scripts. Visual search for potential outliers did not suggest the effect to be driven by outlier scripts. Adding script directionality to the model improved model informativeness (Δ_AIC_ = 4), but the effect of directionality was small and non-significant (β = −0.39, 95% CI [−18, −17]; *df* = 170.11, *t* = −0.43, *p* = 0.96), and not indicative of marked differences between Left-right scripts and Right-left scripts. First halves are more algorithmically complex than last halves, regardless of the script's average complexity and directionality.

**Fig. 8:**
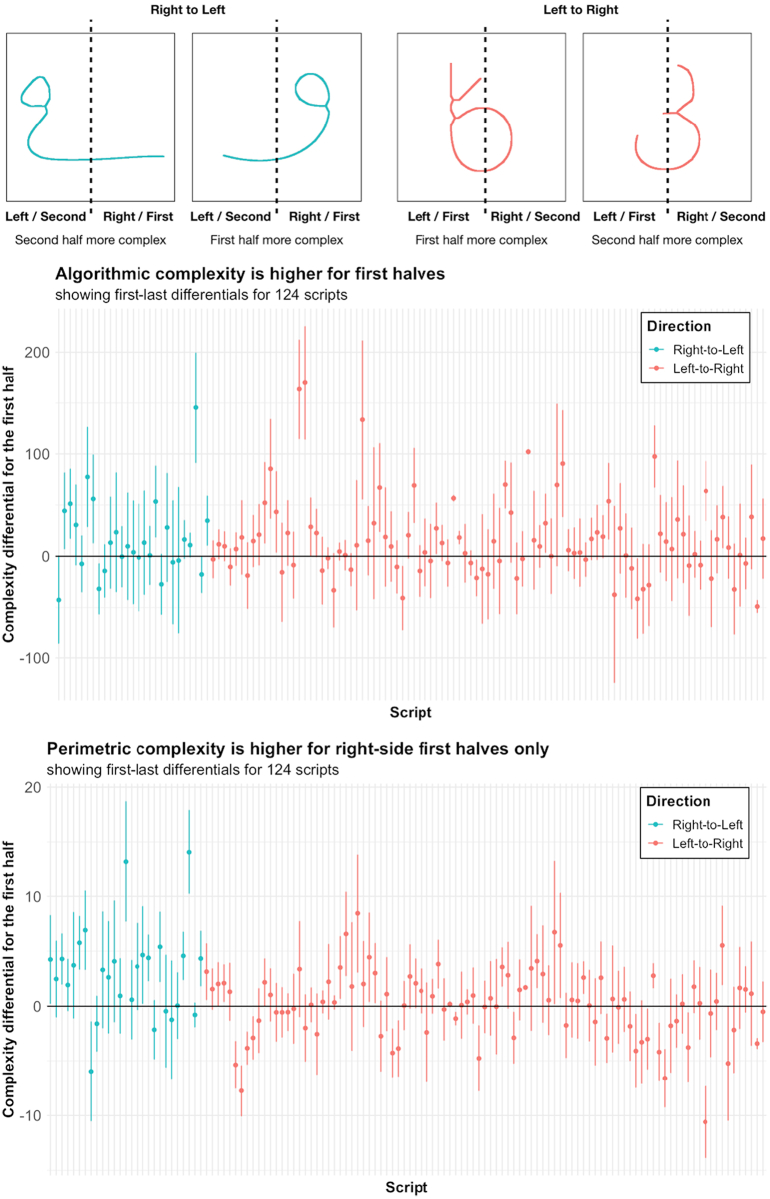
First row: examples of characters from scripts respectively written Left-to-Right (in pink) and Right-to-Left (in blue). The first two characters are borrowed from Psalter Pahlavi [Phlp] (Unicode 10B89 and 10B83), and from Georgian [Geor] (Unicode 010EE and 010D9). For the second and third rows, each point stands for one script, arranged by directionality (Right-to-Left scripts in blue, and Left-to-Right scripts in pink) and alphabetically within each category. Error bars represent 95% confidence intervals. Second row: Algorithmic complexity First-last half differential (AC) by script and writing direction. Third row: average perimetric complexity (PC) First-last half differential by script and writing direction. (For interpretation of the references to color in this figure legend, the reader is referred to the web version of this article.)

For perimetric complexity, the intercept was positive as predicted, but this effect was weak and did not reach significance (β = 0.585; 95% CI [0.001802, 1.1689]; *df* = 98.41, *t* = 1.966, *p* = 0.0521). However, script directionality strongly influenced the first-half / last-half complexity differential. Our model was made more informative by the inclusion of script directionality (Δ_AIC_ = 5), showing a higher first-half / last-half differential for Right-left scripts (effect of directionality: β = 2.82, 95% CI [1.4, 4.2]; *df* = 126.06, *t* = 3.8, *p* = 0.0001). Visual inspection of the data confirmed this: the first halves of characters are perimetrically more complex in Right-left scripts, but not in Left-right scripts (Fig. 8)*.* First halves are slightly more perimetrically complex than last halves, but this is true above all for scripts written from right to left.

#### 3.4.2 Prediction 2: Complexity differentials between character halves depend on order (first vs. last), more than laterality (left vs. right)

We tested this prediction by comparing two models. Each model used character halves as data points, nested according to the character from which they were taken, the script of that character, and the family of that script. We added each half's position in reading order (first or last) as a fixed effect to the first nesting model (order model). In the second nesting model (side model), however, we included each half's laterality (left or right) as a fixed effect. For algorithmic complexity, the prediction was confirmed: the order model was more informative than the side model (Δ_AIC_ = 64). The order model included a clear negative effect for order (order = last, β = −46.3, 95% CI [−48, −44]; *df* = 45,818, *t* = −52.7, *p* < 0.0001), consistent with our previous results on this measure. For perimetric complexity, however, the informativeness advantage in favor of order was not large enough to be interpreted (Δ_AIC_ = 1.3).

#### 3.4.3 Post hoc test, controlling for laterality biases

The fact that our predictions were verified exactly for algorithmic complexity, but only partially so for perimetric complexity, leads us to consider possible biases in our measurement tools. Cutting a character into two halves artificially creates new contours, absent in the original image. Perimetric complexity being, by definition, highly sensitive to contour lengths (unlike algorithmic complexity), this should lead it to overestimate complexity (compared to our other measure). This is a reason to doubt its reliability in the case at hand.

There is also reason to believe that our measurements disagree specifically in ways that confound our predicted effect. Perimetric complexity, compared to our other measure, specifically overestimates the complexity of right-side letter halves (independently of whether they come first or last in writing order). We showed this by considering how the discrepancy between our two measures varies. The discrepancy between perimetric complexity and algorithmic complexity was calculated by normalizing each complexity measure (z-score) for each character half, then subtracting normalized algorithmic complexity from normalized perimetric complexity. Normalization is necessary to ensure that bring our two heterogeneous measures to the same scale so they can be compared. The resulting discrepancy score then served as the dependent variable in a nested regression model. The data points were discrepancy scores for letter halves, nested by letter, script, and writing system type. To these nesting variables we added two fixed effects, one for side (whether the letter half is the right half or the left half), and one for order (whether the letter half is the first half or the last half). The model found a clear effect of side on the discrepancy score (side = right, β = + 0.11, 95% CI [0.09, 0.12]; *df* = 0.0004, *t* = 11, *p* < 0.0001), indicating that perimetric complexity is more likely to be higher than algorithmic complexity on right-side letter halves, controlling for order (not controlling for order does not change this result).

Perimetric complexity is biased against our prediction in a way that algorithmic complexity is not: it is more likely to see right halves as complex. To counterbalance this bias, we needed to test our prediction in a way that is not impacted by disagreements in our measurements concerning the amount of complexity on the right or left side of characters. We did so by testing an alternative prediction: the distribution of complexity within letters is impacted by writing directionality. In other words, we predict that the complexity differential between the left half and the right half is sensitive to writing direction: in scripts written from left to right, the left half / right half differential tends to favor the left half, and vice-versa. This prediction is indifferent to the absolute size or direction of the left half / right half complexity differential, being only interested in how this differential is modulated by the direction of writing in a script. Thus, it tests our hypothesis without being confounded by our measurements' main point of disagreement, i.e., the distribution of visual complexity between the left and right halves of letters.

For each character (in each script, and for each complexity measure), we calculated the Left-right differential, i.e., the complexity of the Left half minus the complexity of the Right half. We then built a null model that predicts this Left half / Right half differential using script and family as a nesting variable. We then included the script's directionality (Right-left as opposed to Left-right) as a fixed variable. Doing so made the model more informative for both complexity measures (Δ_AIC_ = 15 for algorithmic, 14 for perimetric). Both final models showed a negative and significant estimate for the effect of directionality (algorithmic: β = −31.8, 95% CI [−49, −13]; *df* = 170.11, *t* = −3.4, *p* = 0.0007; perimetric: β = −2.9, 95% CI [−4.3, −1.4]; *df* = 126.0, *t* = − 4.0, *p* = 0.0001). The complexity differential in favor of the left half is diminished in scripts written from right to left, i.e., when the right half comes first in reading order. This effect is at least as clear for perimetric complexity as it is for algorithmic complexity. Controlling for each script's overall complexity by adding it as a fixed effect did not change this result, nor did it make either model more informative (Δ_AIC_ = 7/6 in favor of the original model for perimetric/algorithmic measures respectively).

When we test our prediction in a way that is not affected by our measurements' disagreements over the distribution of complexity between the left- and right-side of letters, we find that directionality matters to the distribution of complexity inside letters. The left half is more likely to be the more complex half when it is the first half.

## 4 Discussion

### 4.1 Importance of writing system type and inventory graph size for character complexity

We found the predicted relationship between graph inventory size and character complexity: characters are more complex in large scripts that include numerous characters. We expected this, as characters belonging to larger scripts have to be discriminated from many more characters in order to be recognized. Complexity makes it easier for characters to be distinctive, because it increases the number of ways in which a character can be different from other characters. However, we also found that this positive relationship between graph inventory size and character complexity was only borne out when logographic systems were included in our analyses, ultimately underscoring the highly influential role of typology. In line with this, we found that most of the variance in complexity was accounted for by the type of writing system that a script was mainly used for (e.g., alphabetic, syllabic, etc.).

Contrary to our predictions, the script that a character belonged to did not account for as much variance in character complexity as writing system type did. In previous studies, causality was usually assumed to flow from type to graph inventory size to character complexity. This assumption underpins Chang et al. (2018) evaluation of different measures of character complexity in relation to the different measures' capacity to distinguish between types of scripts. Our results suggest the relationship between type and character complexity may not be mediated by the graph inventory size, as previously assumed (Chang et al., 2016). Rather, writing system type determines both graph inventory size and character complexity, in part by determining the size of the graph inventory. Our result also contradicts Changizi and Shimojo's (2005) claim that character complexity is constant across scripts independently of graph inventory size, implying that different types of writing systems have roughly invariant levels of complexity. Although their results are reproducible when the same exclusions of scripts are made, we also crucially reverse their results when the full range of writing system types and graph inventory sizes is taken into account.

### 4.2 No decrease in character complexity

Overall, there was little evidence of a decrease in complexity over the long-term history of scripts. We put forward two hypotheses derived from our assumption that scripts should manifest a historical decrease in character complexity. Neither were supported. Idiosyncratic scripts were *not* more complex than scripts that were exposed to evolutionary pressures for several centuries. Overall, character complexity did not decrease when parent scripts branched out into descendant scripts. We provide and discuss two possible interpretations of these results: (1) differences in the use of scripts may generate statistical noise, or (2) the decrease in character complexity occurs early and rapidly in a script's history.

Social and cultural factors varying from context to context could have impacted the complexity of scripts' characters. Distinct scripts were used for distinct purposes, and by distinct populations. It is unclear how variation in function would have impacted complexity directly, but it implied different populations of users, ranging from trained scribes to nearly everyone in the population. Higher complexity might have been maintained more easily when scripts were used only by a specialized fraction of the population. Finally, in some cases, writing, and especially handwriting, is made to reflect social belonging, through unnecessary sophistication (Thornton, 1996). Nevertheless, variation in complexity due to function or users can only be expected to have a local influence, i.e., to be circumscribed to the specific contexts and environments in which there is either a narrow function for writing and/or restrictions on who can join the community of users. Any impact that such context-dependent and localized factors may have had on our results can be assumed to be itself localized and context-bound, thus unlikely to bias our results in any systematic way. Still, it may generate enough statistical noise to render any effect in the predicted direction undetectable.

Another possibility would be that compression processes were not captured in the data we gathered and analyzed. This could be the case if the graphic simplification of characters occurred early and rapidly in the history of scripts. This is also in line with the fact that in experimental settings (e.g. (Tamariz & Kirby, 2015)), such effects are known to occur over very short timespans. We know from a more focused study on the Vai script (developed in Liberia during the 19th century), that Vai characters simplified to a substantial degree during the first decades of that script's existence (Kelly et al., 2020).

### 4.3 The distribution of visual complexity inside characters reflects script directionality

For algorithmic complexity, we found, as predicted, that the first halves of letters were more complex than their last halves, regardless of the direction in which the script is written (left-to-right or right-to-left). For perimetric complexity, this first-half advantage was not significant, contrary to what we had predicted. However, we had reasons to believe perimetric complexity to be biased at the level of character halves. Cutting character pictures in half creates new delineations absent from the complete picture, and perimetric complexity is by definition highly sensitive to contour lengths. Furthermore, we showed that our two measures differed in the amount of complexity they measure on the right-side and left-side of letters, quite independently of writing direction. This laterality bias confounds the original tests of our hypothesis. We avoided this bias by considering the influence of direction of reading upon the left-half / right-half complexity differential. We found a clear effect consistent with our prediction for both complexity measures. The direction of reading and writing modulates the distribution of visual complexity within characters, in the predicted direction. The complexity differential between left halves and right halves is increased in Left-right scripts, and decreased in Right-left scripts, clearly and significantly for both complexity measures.

Previous studies had claimed that visual information was unequally distributed within letters, but important divergences exist between authors. The “left-heaviness” of Latin letters has often been noticed (Treiman & Kessler, 2011), but can be interpreted in several ways. Some argue that important information is concentrated on the right-side of (Latin) letters (Kolers, 1969; Shimron & Navon, 1981), others, to the contrary, argue that it is concentrated on the left (Soares et al., 2019). The literature on Chinese characters is more consistent, with several studies finding that the left-side of (roughly) symmetrical characters provides more identification-relevant information (Hsiao & Cottrell, 2009; Tianyin Liu et al., 2018; Tso et al., 2014), an effect possibly mediated by reading direction (mostly left-to-right in contemporary Chinese) (Chung et al., 2017). Beyond these two famous scripts (and possibly a few others like Hebrew), the study of information distribution within letters remains unstudied for most literate cultures, and the heterogeneity of methods and measurements allows contradictory positions to persist.

Insofar as visual complexity can be interpreted as a proxy for visual information, our study confirms, on a large observational dataset, the intuition that the majority of experimental studies had gotten from studying a few scripts. The direction of reading and writing drives the distribution of complexity inside letters: left halves are more likely to be more complex when they come first.

From a cultural evolution point of view, this result supports the burgeoning body of work studying the ways in which letter shapes fit subtle cognitive and perceptual biases. Interestingly, here, the cognitive bias in question is not derived from the structure of natural scenes (as in Changizi et al., 2006), and neither is it rooted in fundamental neural biases (like the preference for cardinal orientations in Morin, 2018). Rather, the shape of letters adapts to a culturally induced cognitive bias, which finds its roots in the motor and visual habits created by reading and writing directions.

### 4.4 Limitations and future directions

The current study has several limitations. First, the images we used to analyze scripts were idealizations (as any representation of a script must be). They abstract away a great deal of internal variation due to time, space, and differences between writers, etc. There is also a need to study writing systems on their own, through their own chronological trajectory, which this study was not meant to fulfil (see Kelly et al., 2020 for an example: the Vai syllabary of Liberia).

Second, the visual complexity of individual characters is only one of many possible ways to consider complexity in scripts. Future research could address other types of complexity and their evolution, such as set-level complexity (i.e., how compressible is the whole set of characters included in a script), or as the inventory of patterns or features are re-used by different characters in the same script (Bennett, Gacs, Li, Vitanyi, & Zurek, 1998; Vitányi, Balbach, Cilibrasi, & Li, 2009). This may allow us to capture constraints related to feature extraction, i.e., visual processing which treats some parts of characters as separate units, and to discriminability between characters (Mueller & Weidemann, 2012).

Third, character complexity also depends on the way characters are combined and occur in real world settings. The visual complexity of scripts *in use* differs from the complexity measured on their characters independently from one another. When used alongside simpler characters, higher complexity characters can benefit from a processing advantage, for instance during visual search (Bernard & Chung, 2011; Chanceaux, Mathôt, & Grainger, 2014). More complex characters would, in that case, have more different features available for use during visual search, helping to distinguish them from other characters.

In this study, we focused on the visual aspect of characters, and how they are perceived, i.e., how they are recognized as characters by a reader. Nevertheless, characters also have to be produced. For most of the history of writing, characters were hand-written. Although more complex characters would, overall, be more effortful to produce than simpler ones, some shapes may be easier to produce than others. Changes related to motor production may also have an impact on the graphic complexity of characters (see Parkes, 2008 on cursivization). Motor-program based estimates of complexity (Lake, Salakhutdinov, & Tenenbaum, 2015) could offer computational options for such future research.

The complexity of written characters is a fundamental aspect of their legibility, determining their recognizability and the cognitive effort required to process them (Pelli et al., 2006). Understanding it properly requires us to grapple with a great diversity of writing systems, a daunting task for which research sometimes falls short. An excessive concentration on a few important scripts (like Latin, Chinese, or Arabic), the use of idiosyncratic metrics tailor-made for some scripts but inapplicable to others, or the neglect of Galton's problem, are biases that can prevent us from noticing important patterns. As a result, the literature's conclusions on letter complexity can seem contradictory. For instance, complexity is constant across scripts for some studies, while modulated by writing system type for others. This study attempted to provide a methodologically sound and comprehensive overview of these issues. Our results reconcile discrepancies between previous studies and uncover surprising patterns. Two key findings are the unrivalled importance of typology in driving character complexity, and the fact that the distribution of visual information within characters follows reading order. These general patterns call for more detailed investigations of the cultural evolution of writing systems, based on more focused script-specific datasets (e.g. Kelly et al., 2020).

## Footnotes

1 All scripts included in our dataset are associated to unique 4-letter identifiers in the ISO15924 (e.g., [Latn] for the Latin script), which we use here to avoid confusion.

2 The one featural system included in the present study is Korean Hangul [Hang]. In featural systems, the shape of characters correlates with the language's features. In Hangul, the shape of characters was designed to resemble the shape taken by the mouth to produce the corresponding sounds.
